# A SNP Mutation of SiCRC Regulates Seed Number Per Capsule and Capsule Length of *cs1* Mutant in Sesame

**DOI:** 10.3390/ijms20164056

**Published:** 2019-08-20

**Authors:** Libin Wei, Chun Li, Yinghui Duan, Wenwen Qu, Huili Wang, Hongmei Miao, Haiyang Zhang

**Affiliations:** Henan Sesame Research Center, Henan Academy of Agricultural Sciences, Zhengzhou 450002, China

**Keywords:** sesame, seeds number per capsule, capsule length, CRABS CLAW, hormone

## Abstract

Seed number per capsule (SNC) is a major factor influencing seed yield and is an important trait with complex gene interaction effects. We first performed genetic analysis, gene cloning, and molecular mechanism study for an EMS-induced sesame mutant *cs1* with fewer SNC and shorter capsule length (CL). The mutant traits were due to the pleiotropism of a regressive gene (*Sics1*). Capsule hormone determination showed that five out of 12 hormones, including auxin indole-3-acetic acid (IAA), had significantly different levels between wild type (WT) and mutant type (MT). KEGG pathway analysis showed that plant hormone signal transduction, especially the auxin signal transduction pathway, was the most abundant differentially expressed signaling pathway. After the cross-population association and regional genome screening, we found that three homozygous loci were retained in *cs1*. Further analysis of these three loci resulted in the identification of SiCRC as the candidate gene for *cs1*. SiCRC consists of seven exons and six introns encoding 163 amino acids. The SiCRC in *cs1* showed a point mutation at intron 5 and exon 6 junction, resulting in the splice site being frame-shifted eight nucleotides further downstream, causing incorrect splicing. Taken together, we assumed the SNP mutation in SiCRC disrupted the function of the transcription factor, which might act downstream of the CRC-auxin signal transduction pathway, resulting in a shorter CL and less SNC mutation of *cs1* in sesame. Our results highlight the molecular framework underlying the transcription factor CRC-mediated role of auxin transduction in SNC and CL development.

## 1. Introduction

Sesame (*Sesamum indicum* L., 2n = 26) is an important oilseed crop with high nutrition and oil quality [1]. Sesame is a very rewarding crop because of its high sale price. However, the seed yield capacity of sesame was very low compared to other oilseed crops. Therefore, breeding high-yielding sesame varieties is one of the key objectives for sesame production [2].

To develop high-yielding genotypes, knowledge regarding the nature and magnitude of gene action governing the inheritance of yield and yield components are pre-requisite to the design of efficient breeding programs [3,4]. Though seed yield is known to be a complex trait, it can be improved through improvement of yield contributing factors [5,6,7]. Seed number per capsule (SNC), which is one of the three major factors influencing seed yield, has been considered as an important trait with complex gene interaction effects [8,9]. The more SNC, the more seed yield. Moreover, SNC generally has a positive correlation with the capsule length (CL) [10,11]. Therefore, a detailed understanding of the genetic basis and molecular regulation mechanism of SNC and CL traits was very important for the breeder on developing high-yielding sesame varieties in the future.

The inheritance and nature for sesame SNC and CL traits has been studied by many scholars. Most scholars pointed out that both of them are complex traits with complex gene interaction effects in various parent combinations in different ecological regions [9,12,13]. However, the molecular mechanism and associated molecular genetic information underlying these important yield-related traits have not yet been systematically explored. Until now, only a few studies on the QTL mapping have been reported. Wu et al. (2014) constructed a high-density genetic map of sesame and identified three QTLs related to the seed number per capsule trait and five QTLs related to the CL trait [6]. In addition, through the genome-wide association studies method, Zhou et al. (2018) identified one seed number per capsule-related QTL and four CL-related QTLs [14]. The accurate target genes for the seed number per capsule and CL traits have not been detected until now. Therefore, cloning the *cs1* gene may provide new details regarding the molecular mechanism regulating sesame seed number per capsule and CL development.

In recent years, with the rapid development of next-generation sequencing technology (NGS) and bioinformatics tools, the linkage and association mapping methods have been widely used for rapid detection of QTLs and candidate genes [15,16,17,18,19,20,21]. Furthermore, mutants have been proven to be very important materials for the study of the inheritance and molecular basis for complex agronomic traits [16,22,23,24,25], especially for diploid cultivated sesame with the smaller genome size of 354 Mb [2]. A sesame mutant simultaneously affecting the traits of seed number per capsule and CL named Yuzhi sn (*cs1* line) was created from the sesame cultivar Yuzhi 11 (selected as the reference genome for the Sesame Genome Project) using EMS mutagenesis in 2010. The *cs1* mutant line has a low SNC and short CL and cannot be affected by environmental factors.

Here, we performed an integrated analysis of the transcriptome, hormonome, and genome to (1) analyze the genetic background of the *Sics1* mutant, (2) identify target gene *Sics1* in sesame, and (3) exploit the mutagenesis characteristic and gene regulatory pathways of the *Sics1* mutant in sesame. The findings provide new frameworks regarding the molecular mechanism regulating sesame seed number per capsule and CL development.

## 2. Results

### 2.1. Phenotypic Comparison

**Figure 1:**
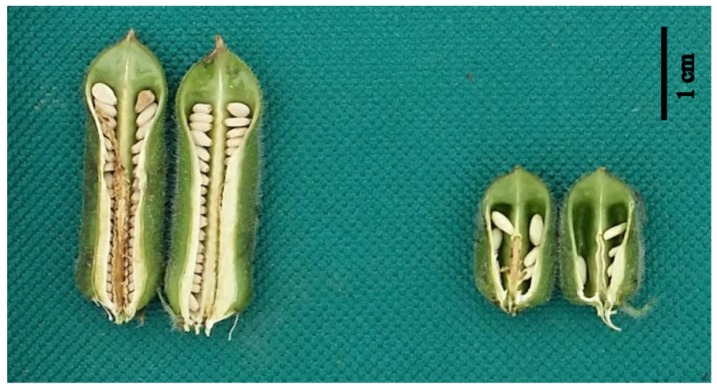
Phenotypic comparison of the capsule and seeds between Yuzhi 11 (left) and *cs1* (right).

Phenotypic observation showed that there was a significant difference in the agronomic traits of CL and SNC between *cs1* mutant type (MT) and the Yuzhi 11 wild type (WT) (Figure 1, Table S1, Figure S1). Compared with the 3.3 ± 0.3 cm CL in Yuzhi 11, the CL of *cs1* shorten to 1.6 ± 0.2 cm (*p* < 0.01). Meanwhile, the SNC of *cs1* was reduced to 8 ± 2 compared with Yuzhi 11 with 72 ± 4 seeds per capsule (*p* < 0.01).

### 2.2. Inheritance Analysis

In order to clarify the inheritance of *cs1* genotype in sesame, we constructed F1 hybrids, BC1 and F2 populations of cross combination of mutant *cs1* × Yuzhi 11. The F1 generations from reciprocal crosses between *cs1* and Yuzhi 11 both displayed the normal CL and seed number per capsule just as the Yuzhi 11 wild type (Table 1), which revealed recessive gene control for the two mutant traits. In addition, these two mutant traits always occur simultaneously in the BC1 and F2 populations, which indicate the pleiotropism of a single mutant gene.

In BC1 population, the segregation ratio of the MT and the WT traits fit the expected 1 (*cs*):1 (*CS*) ratio (Table 1). Additionally, the segregation ratio of the wild and mutation types in the F_2_ populations fit the expected ratio of 3(*CS*):1(*cs*). Chi-square tests (*p* > 0.05) proved that the segregation of the mutant traits in mutant *cs1* fit the Mendelian inheritance mode. The results confirmed that the mutant traits were controlled by a regressive gene. Thus, we annotated the shorter CL and fewer SNC locus in mutant *cs1* and Yuzhi 11 (WT) as *Sics1* and SiCS1, respectively.

### 2.3. Plant Hormone Determination

**Figure 2:**
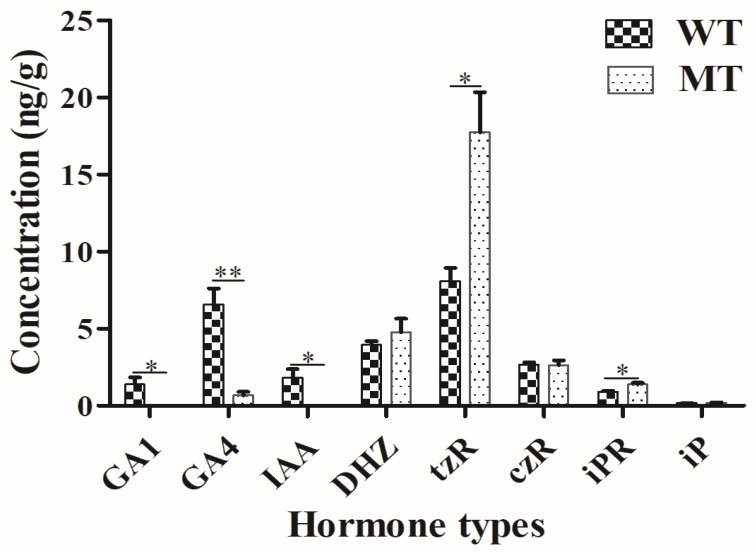
Comparison of hormones content between WT and MT samples. The numbers of concentration are mean values with their respective standard deviation. Significant differences were marked with up to asterisks. * (*p* < 0.05), ** (*p* < 0.01).

To determinate GA (GA1, GA3, GA4, and GA7), IAA and CKs (tZ, cZ, DHZ, tzR, czR, iPR, and iP) content of the two kinds of capsule samples with 3 wild types (WT) and 3 mutant types (MT), LC–MS/MS were used. Four hormones (GA3, GA7, tZ, and cZ) could not be detected in these samples. Five (GA1, GA4, IAA, tzR, and iPR) out of these 12 hormones showed significantly different levels between WT and MT samples (Figure 2). More specifically, the levels of GA1, GA4, and IAA were higher in WT than in MT. In contrast, the levels of tZR and iPR were lower in WT than in MT.

### 2.4. RNA-Seq Analysis

Two kinds of capsule samples were sequenced with the Illumina sequencing platform. A total of 63.0 Gb of clean data was obtained with the average 10.5 Gb per sample (Table 2). The percentages of unique mapping reads matching the sesame reference genome (version 3) were more than 91.0% (Table 2).

The DEGs were analyzed further between WT and MT samples. Pearson correlation analysis showed good sample reproducibility for both WT and MT with R^2^ ≥ 0.933 (Figure S2). A total of 480 DEGs were detected between WT and MT samples, with 150 and 330 genes down- and up-regulated in the MT sample, respectively (Figure S3).

**Figure 3:**
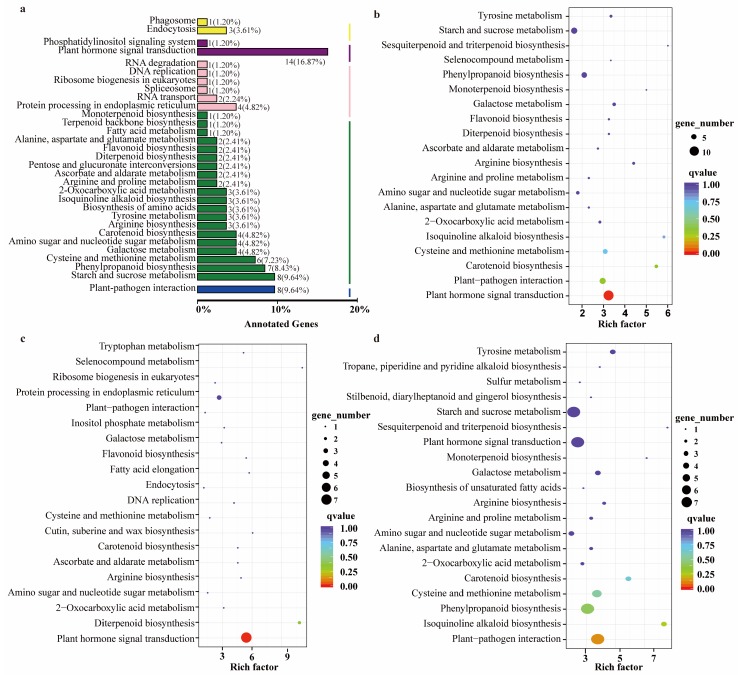
KEGG pathway enrichment for DEGs. (**a**,**b**): The top enriched KEGG pathway for all DEGs. (**c**,**d)**: The top enriched KEGG pathway of down-regulated DEGs and up-regulated DEGs, respectively.

To further understand the enriched pathways for the mutant traits, the KEGG database was used to effectively analyze the DEGs, and 56 pathways were identified (Table S2). Among these KEGG pathways, plant hormone signal transduction, starch and sucrose metabolism, plant-pathogen interaction, phenylpropanoid biosynthesis, and cysteine and methionine metabolism were the five most significantly enriched KEGG pathways (Figure 3a,b). What is noteworthy is that more than 70% of the DEGs in the plant hormone signal transduction pathway participated in the auxin signal transduction pathway (Table S3). More specifically, a total of 24 pathways were identified for down-regulated DEGs, and plant hormone signal transduction was the most abundant differentially expressed signaling pathway with rich factor >5.27 (Figure 3c). Moreover, all of the down-regulated DEGs in plant hormone signal transduction involved in auxin signal transduction pathway (Table S3). As for the pathways related to the up-regulated DEGs, plant hormone signal transduction also was one of the most abundant differentially expressed signaling pathways, as well as starch and sucrose metabolism, phenylpropanoid biosynthesis and plant-pathogen interaction pathways (Figure 3d).

### 2.5. Sics1 Gene Identification

To efficiently locate the gene locus of the mutant trait, we carried out the cross-population association mapping and genome-wide screening strategy. The two parents (*cs1* × 15TP1783) and their 131 F_6_ RILs were sequenced using an Illumina HiSeq 2500 sequencing approach. In total, 766.66 Gb of raw data was obtained with the average genome coverage of 16.17-fold per sample (Table 3). The mapped reads of each sample were aligned to the sesame reference genome (var. Yuzhi 11, PRJNA315784) for SNP/InDel calling. A total of 325,005 unique SNPs was found in the two parents (Table 3).

**Figure 4:**
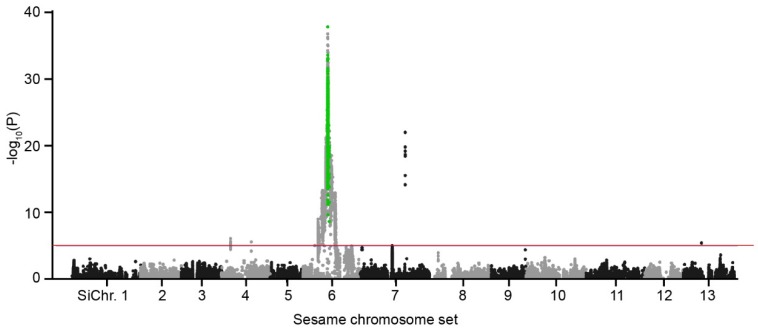
Cross-population association mapping of *Sics1* gene locus in sesame. Manhattan plot of SNP/InDel association mapping of the MT is performed using a RIL population. The peak of -log 10 (*p*) is located on *Si*Chr. 6. After screened using the genome variants data, 156 variants (green dots) are retained as the candidate markers associated with the MT. Note: each dot (black, grey and green) represents a SNP/InDel variant. Red line refers to cutoff used in association mapping.

Subsequently, all the unique SNPs were applied for variants screening in the 131 individuals of the RIL population. After filtering, 496,486 SNP/InDel variants were detected for Joint calling. According to the reference sesame genome (Yuzhi 11) and the nomination of the sesame chromosome set [23,26], a total of 423,138 SNP/InDel variants were plotted in 13 chromosomes (Figure 4). Association mapping results showed that the variant site (C11_4680332) with the lowest *p*-value (5.43E^−51^) was located on the contig 11 of *Si*Chr.6. To screen the target variants from the target contig, we chose the up- and down-stream 200 kb flanking sequences of C11_4680332 as the target interval linked to the mutant trait (*cs1*).

The interval C11 between C11_4502464 and C11_4880247 markers contained 2143 variants with the *p*-value variation of 5.43E^−51^ to 0.997 (Table S4). Then we filtered these detected 2143 SNP/InDel variants using the regional genome variant data of 620 sesame accessions (wild type). For the 620 accessions, 4738 SNPs/InDels existed in the region of C11_4502464 and C11_4880247 (Table S5). Genomic variant screening results showed that 156 variant loci (green dots in Figure 4) were retained in the target interval (Table S6). Further study conclusively showed only three out of 156 SNP/InDel loci in this candidate region were homozygous loci and were specially retained in *cs1*. Fortunately, of the three variants, two variant positions were located at the intergenic region and one at a splice acceptor site (Table S7). So, the splice acceptor site mutation (C11_4747318, G→T) was considered as the candidate mutant locus for *cs1*.

At the same time, to confirm this SNP site in *cs1* genotype, we designed the AS-PCR molecular marker (SicsSNP) (Table S8) and screened the test population including 700 F_7_ RILs of the combination of *cs1* and 15TP1783, and 300 sesame germplasm accessions with WT capsule (Figure S4). PCR screening results proved that the SicsSNP alleles entirely accorded with the phenotype in the test population. Then the gene containing the SicsSNP locus was named *Sics1* and regarded as the most likely candidate gene, and the SNP is responsible for the *cs1* mutant phenotype.

### 2.6. Structure Analysis of SiCS1 Gene and Homolog Comparison

**Figure 5:**
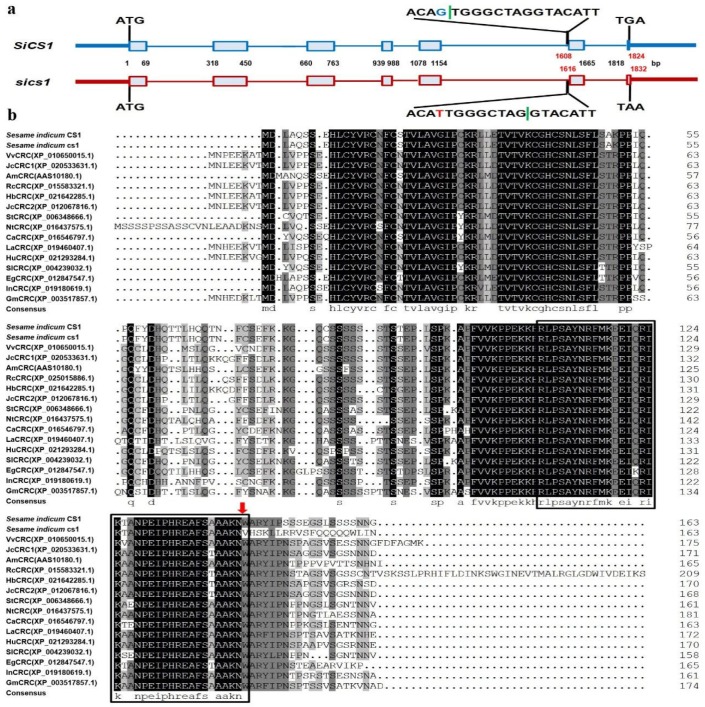
Structure analysis and sequence comparison of SiCS1 and *Sics1* homologs in sesame and other plant species. (**a**): Structure comparison of SiCS1 and *Sics1*. (**b**): Protein sequence comparison of SiCS1 and *Sics1* homologs in sesame and other plant species. The box in b labeled HMGB-UBF_HMG-box conserved domain. The red arrow indicates the mutation site. Note: the green vertical lines refer to the splicing point. The blue G and red T refer to SNP variant between SiCS1 and *Sics1.*

With the aid of the reference genome information of var. Yuzhi 11, we designed the primer pairs and amplified the entire cDNA and DNA sequences of the SiCS1 allele (Table S9). Sanger sequencing and gene alignment results proved that the full DNA sequence length of SiCS1 gene (NCBI accession no. KY649621.1) in Yuzhi 11 was 1824bp consisting of seven exons and six introns encoding 163 amino acids (Figure 5). For *cs1*, the SNP mutation of G_1608_T occurred at intron 5 and exon 6 junction of the gene, which caused the wrong splicing (splicing point retruded eight nucleic acids at 1616th locus) in SiCS1 after the 143rd amino acid (Figure 5), and changed the full genome length of 1824 bp in SiCS1 to 1832 bp in *Sics1*. Non-redundant (NR) protein annotation results revealed that the SiCS1 gene was identified to encode a transcription factor CRABS CLAW-like protein (CRC). BlastP analysis indicated that there was a homolog gene SiCS2 (XM_020697194.1, annotated as a protein CRABS CLAW-like) with 96.34% identities to SiCS1 in sesame (Figure S5). Moreover, the SiCS1 had a high resemblance to the CRC homologs in the other species, as the resemblance rate varied from 73.62% (LaCRC) to 80.98% (VvCRC) (Figure 5b). Compared with these CRC homologs, the splicing mutation of *Sics1* occurred in the conserved sequence region (Figure 5b). Of the 15 homologs in the other 14 plants, CRC homologs of *Erythranthe guttata* displayed the closest relationship with SiCS1 protein (Figure S6).

### 2.7. Expression Profiles of SiCS1 in Sesame

**Figure 6:**
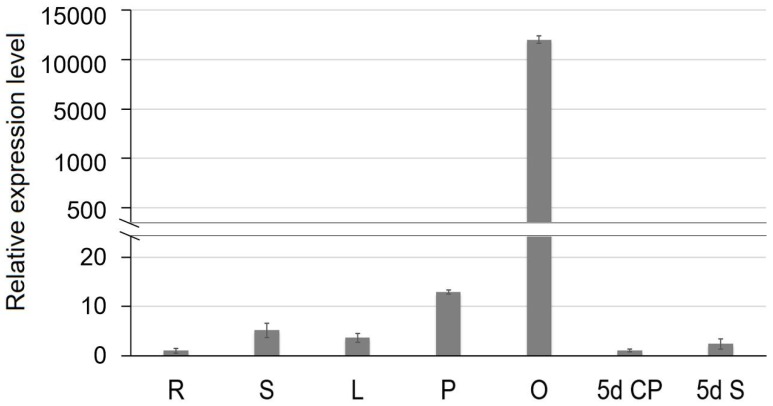
Expression analysis of SiCS1 in different tissues of Yuzhi 11 by qRT-PCR. R root, S stem, L leaf, P petals with stamens, O ovary at the flowering day, 5d CP 5 days capsule peel after flowering, 5d S 5 days developing seeds after flowering.

To reveal the expression profiles of SiCS1 in sesame, we designed the real-time PCR primer and monitored the transcription level of SiCS1 in root, leaf, stem, bud, ovary, capsule peel five days after flowering, and developing seeds five days after flowering in Yuzhi 11 using real-time quantitative PCR (qRT-PCR) (Table S9). The results displayed that SiCS1 genes specifically expressed in the tissues of the ovary (relative expression level was more than 11,982.0) but were barely detected in other tissues (relative expression level was less than 13.0) (Figure 6). The morphogenesis of the ovary also initiated the first stage of the capsule, and the expression of SiCS1 affecting this organ also influenced capsule development.

## 3. Discussion

SNC and CL are important traits for sesame yield [8,10,11]. However, the molecular genetic information and mechanism underlying these yield-contributing traits have not yet been systematically explored. Here, a sesame mutant *cs1* with both SNC and CL simultaneous mutation had been created by artificial EMS mutagenesis from HAAS for the first time.

According to the results of the hormone determination, five out of these 12 hormones showed significantly different levels between WT and MT samples including three hormones (GA1, GA4, and IAA) higher and two hormones (tZ and cZ) lower in WT than in MT. These phenomena indicated the formation mechanism of the phenotype mutant in *cs1* was complex, and many factors were responsible for the mutant phenotype.

Furthermore, RNA-Seq was used to study the molecular mechanism of the phenotype mutant in *cs1*. DEGs analysis showed that the number of down-regulated genes and pathways was less than that of up-regulated genes and pathways. Fortunately, according to the results of KEGG pathway analysis, the plant hormone signal transduction was the most abundant differentially expressed signaling pathway, and all of the down-regulated DEGs in plant hormone signal transduction involved in the auxin signal transduction pathway, which was in accordance with the result of the IAA hormone determination. Thus, we assumed that the change of IAA level may be mainly responsible for the mutant phenotype of *cs1*.

Moreover, based on the cross-population association mapping, genomic variants screening, bioinformatics analysis, and AS-PCR molecular marker validation technologies, the CRABS CLAW (CRC) gene in sesame with a SNP mutation at intron 5 and exon 6 junction was identified as the target gene for its incorrect splicing after the 143rd amino acid. Previous research in other plants showed that CRC, as a member of the YABBY transcription factor family, plays a vital role in nectary and carpel development [18,27,28,29,30].

In our study, the CL and SNC mutant traits were due to pleiotropism of a regressive gene (*Sics1*), which was consistent with the phenotype mutation in the *Arabidopsis thaliana crc* mutant [27,31]. The same pleiotropic effects on silique length and seed number were also observed in *Atcrc* plants. The mature silique is shorter and the number of seeds per gynoecium is also strongly reduced in the CRC mutation [27,31]. More importantly, through comparing previous gene mapping results on CL and SNC traits from linkage mapping and genome-wide association studies with our result [6,14], the aimed gene identified in this study was a novel gene for there was no overlap with the previously mapped locations (Table S10).

Based on our expression profiling and DEGs pathway analysis, we considered plant hormone signal transduction as the pathway acting downstream of CRC. Since the role of the pathway in sesame SNC and CL development is largely unknown, and the most abundant differentially expressed signaling pathway was identified in these pathways, we focused on the auxin signal transduction as a putative direct pathway target of CRC. Although the pathway that functions downstream of CRC to influence auxin expression is not yet fully understood, CRC controls auxin transport, which has been reported by Yamaguchi et al. [30]. In their study, they also pointed out that CRC controls auxin homeostasis and establishes auxin maxima [30]. In addition, Alvarez and Smyth (2002) pointed out that strongly localized auxin flux may assist the early growth of progenitors of the placentae and septum. Also, carpels may be in a floral-specific developmental program of auxin-induced vascularization [31].

Thus, we assumed that the incorrect splicing SNP of SiCRC in the mutant *cs1* significantly affected the expression of the genes involved in the auxin signal transduction pathway. Then, in order to remedy this defect, a series of genes for physiology, energy, and substance synthesis began to have up-regulated expressions in the mutant *cs1*, such as the genes participating in the pathways of plant hormone signal transduction, starch and sucrose metabolism, and phenylpropanoid biosynthesis, and so on. Although a higher number of up-regulated genes participated in many different pathways, the mutant phenotype cannot be remedied. In addition, because our RNA-Seq analysis identified many DEGs involved in the plant hormone signal transduction pathway, it is also possible that CRC has more downstream targets that jointly control hormone transport and metabolism (biosynthesis, conjugation, and degradation) at multiple levels. Therefore, future investigations and functional studies are needed to understand how *Sicrc* disrupts hormone homeostasis and leads to the change of these phenotypes.

## 4. Materials and Methods

### 4.1. Plant Materials

A shorter CL mutant *cs1* with fewer SNC was induced from a subline (90-1) of Yuzhi 11 using EMS mutagenesis and was self-pollinated more than four generations before the genetic analysis. The F_1_, BC_1_, and F_2_ populations of the cross between the mutant *cs1* and the wild type Yuzhi11 were used to investigate the inheritance characterization of mutant traits. The 131 out of 831 F_6_ RILs of the cross between *cs1* and 15TP1783 with longer CL and more SNC were used for genome re-sequencing and gene locus detection in 2015. At the same time, to reveal the gene regulatory pathways of *cs1* mutant, two kinds of capsule peel tissues (30 F_6_ RILs mentioned above with the mutant type like *cs1* traits and 30 F_6_ wild-type RILs with normal CL and SNC) were collected at the 4 (± 1) days after flowering for RNA-Seq analysis and plant hormone (gibberellins, auxin, and cytokinins) determination analysis. Furthermore, the rest of the 700 F_7_ RILs of the combined population of *cs1* and 15TP1783, and 300 germplasm accessions with normal CL and SNC trait were cultured at Yuanyang experimental station for phenotype observation and leaf collection in 2016 and were used for the target gene verification by the traditional PCR method. All the above germplasm and populations were available from Henan Sesame Research Center, Henan Academy of Agricultural Sciences (HSRC, HAAS) (Zhengzhou, China). Young leaf tissues of the above accessions and population progeny were collected, immersed in liquid nitrogen and frozen at −80 °C for the following studies.

### 4.2. Genomic DNA and RNA Extraction and Sequencing

Genomic DNA was extracted from young leaves of the mapping population using DNeasy Plant Mini Kits (QIAGEN, Hilden, Germany). Total RNA was extracted from capsule tissues following the use of TRIzol reagent (Invitrogen, Shanghai, China) according to the manufacturer’s instructions. Both RNA-Seq and genome re-sequencing were carried out on an Illumina HiSeq 2500 platform following the manufacturer’s protocol in Beijing Biomarker Technologies Co. Ltd (Beijing, China). Each kind of RNA-Seq were performed with three biological replicates.

### 4.3. Plant Hormone Determination

Endogenous hormone determination of two kinds of capsule samples was performed using ultra-performance liquid chromatography LC–MS/MS. The extraction and purification of approximately 100 mg of each sample were performed according to the method supplied by Shanghai Applied Protein Technology Co. Ltd (Shanghai, China). Three biological replicates were analyzed for each sample. The internal standards of gibberellins (GA), auxin indole-3-acetic acid (IAA) and cytokinins (CKs) were added to each sample. Then, each hormone such as 4 GAs (GA1, GA3, GA4 and GA7), IAA and 7 CKs (trans-zeatin (tZ), cis-zeatin (cZ), dihydrozeatin (DHZ), trans-zeatin riboside (tzR), cis-zeatin riboside (czR), isopentenyladenosine (iPR) and isopentenyladenine (iP)) was quantified by comparing its peak area with the peak area of its respective internal standard using *MultiQuant*™ software (version 3.0.2, Sciex, Framingham, MA, USA).

### 4.4. Sequencing Data Analysis

All raw reads obtained from the Illumina HiSeq2500 platform were filtered using Trimmomatic 0.33 [32]. As for RNA-Seq data, clean reads were mapped to the reference genome sequence using Tophat2 [33]. StringTie was used for reading the assembly and quantification analysis [34]. Gene expression levels were measured using the fragments per kilobase of transcript per million mapped fragments (FKPM) method [35]. Differential expression analysis between two groups of capsule traits was performed using the DESeq R package [36]. The resulting *p*-values were adjusted using the Benjamini and Hochberg approach for controlling the false discovery rate (FDR) [36]. Genes identified by DESeq with FDR < 0.01 and fold change (FC) > 2 were defined as differentially expressed genes (DEGs). Functional annotations of the DEGs were performed and searched against the NR, Swiss-Prot, KEGG (Kyoto Encyclopedia of Genes and Genomes) databases. Metabolic pathway assignments were carried out based on the KEGG orthology database (http://www.genome.ad.jp /kegg/) using KOBAS2.0 [37,38].

Alignment of re-sequencing data to the reference genome was performed using BWA 0.7.15 with the default settings described by Li and Durbin [39]. Putative SNPs and InDels were screened using Genome Analysis Tool Kit (GATK3.7) packages according to GATK joint calling best practice [40]. All the variants from all the 131 sequencing samples were filtered according to the following high quality (high-confidence) criteria: minimal variant count ≥100 and minimum frequency of 0.1 (the minimum frequency of the minority polymorphisms for the site). The fine sesame genome data of Yuzhi 11 (version 3.0, Zhengzhou, China) was used as the reference genome in this study [7]. The chromosome position of the target gene was determined according to the integration of genome assembly data and the chromosome annotation information [7,26].

### 4.5. Statistical Significance and Candidate Gene Location

Association analysis of all the above variants with the phenotype information of SNC and CL in the RIL population was performed using the GLM (general linear model) model in TASSEL 5.0. The target locus was defined according to the specific interval with the lowest *p*-value. Home-made scripts were used to screen the specific SNPs and InDels of the target interval using the genomic variants data of 620 sesame accessions (regional data at www.sesamum.org).

The candidate SNP/InDels were transformed into PCR-based markers using the Primer Premier 5.0 program (http://www.premierbiosoft.com/prierdesign/index.html) according to the method of Wei et al. [41]. PCR reaction was carried out on a PTC-225 machine (MJ Research, Waltham, MA, USA) according to the description of Zhang et al. [23]. All the PCR products were electrophoresed in 8% non-denaturing polyacrylamide gels and visualized via silver staining [42].

### 4.6. Cloning and Annotation of SiCS1 Gene

To clone the entire gDNA and cDNA sequences of SiCS1 alleles in sesame, the primer pairs were designed using Primer Premier 5.0. PCR amplification was carried out on a PTC-225 machine (MJ Research, Waltham, MA, USA). The PCR products were individually gel-purified for Sanger sequencing with three replications. Non-redundant (NR) protein and KEGG annotations for candidate genes were obtained using BLASTP and BLAST2GO, respectively.

### 4.7. SiCS1 Homolog Detection and Phylogenetic Analysis

BLASTP was applied to screen *Sics1* homolog(s) in Yuzhi 11 reference genome. The amino acid sequences of SiCS1 and *Sics1* were aligned with the homologs in *Vitis vinifera* (Vv), *Jatropha curcas* (Jc), *Antirrhinum majus* (Am), *Erythranthe guttata* (Eg), *Ricinus communis* (Rc), *Solanum tuberosum* (St), *Nicotiana tabacum* (Nt), *Capsicum annuum* (Ca), *Lupinus angustifolius*(La), *Ipomoea nil* (In), and *Glycine max* (Gm), respectively, using DNAMAN (http://www.lynnon.com/pc/framepc.html). All the above homologs information was downloaded from NCBI dataset. A phylogenetic tree was constructed based on the above orthologs using the MEGA5 program [43].

### 4.8. Expression Profile Assay of SiCS1 Gene

Total RNA was extracted from different tissues including root, stem, leaf, petals with stamens, ovary at the flowering day, capsule peel 5 days after flowering, and developing seeds 5 days after flowering using TriZOL Reagent (Invitrogen, Shanghai, China). The primer pairs of SiCS1 gene for quantitative real-time PCR (qRT-PCR) analyses were designed using the Primer Premier 5.0 program. Real-time PCR reaction was performed on a Master cycler realplex (Eppendorf, Hamburg, Germany) according to the standard method of Wei et al. [44]. Transcript amount of SiCS1 gene was normalized against the *β-tubulin* gene and compared using the ∆∆Ct method [44]. 

## 5. Conclusions

In the present study, an integrated analysis of the sesame transcriptome, hormonome, and genome revealed an incorrect splicing mutation of SiCRC in the auxin signal transduction pathway is simultaneously responsible for two important yield-contributing traits (SNC and CL) in sesame. This study may lay a foundation for further study on the molecular mechanism of the characteristics for SNC and CL in sesame.

## Figures and Tables

**Table 1:** Inheritance analysis of the Sc and sc1 phenotypes in sesame.

Population Type	CS Phenotype No.	cs Phenotype No.	Expected Ratio	χ^2^ Value
(sc1 × Yuzhi11) F1	42	0	-	-
(Yuzhi11 × sc1) F1	46	0	-	-
BC_1_	181	173	1:1	0.18
F_2_	584	206	3:1	0.49

**Table 2:** Summary for the transcriptome of MT and WT samples.

Category	MT1	MT2	MT3	WT1	WT2	WT3
Obtained Clean Pair-end Reads (million)	30.6	36.,3	31.7	32.5	35.3	43.9
Obtained Clean Bases (Mbp)	9159.9	10,873.4	9498.5	9749.9	10,588.4	13,147.7
GC Content	46.69%	47.78%	46.89%	46.81%	46.67%	46.66%
Q30 %	94.14%	94.18%	94.37%	94.05%	94.10%	93.86%
Mapped Reads (million) (%)	58.1 (94.81%)	68.5 (94.29%)	60.1 (94.72%)	61.7 (94.78%)	66.9 (94.71%)	83.1 (94.70%)
Unique Mapped Reads (million) (%)	56.9 (92.92%)	66.1 (91.05%)	58.9 (92.78%)	60.5 (92.87%)	65.6 (92.82%)	81.3 (92.67%)
Multiple Map Reads (million) (%)	1.2 (1.89%)	2.4 (3.24%)	1.2 (1.94%)	1.2 (1.90%)	1.3 (1.88%)	1.8 (2.03%)

**Table 3:** Genome sequencing information of the mapping population for mutant trait.

Sample Name	Raw Read Number (Million)	Coverage (×) ^a^	Ratio of High-Quality Reads		Variant Loci Number	Unique LOCI ^c^
			Q ≥ 20	Q ≥ 30		
Mutant *cs1* (P_1_)	54.2	22.97	96.12	91.65	284,753	124,578
15TP1783 (P_2_)	57.6	24.39	95.90	91.35	360,602	200,427
131 F_6_ progeny ^b^	4999.3	2118.35	96.72	92.97	/	/
Total	5111.1	2165.72	96.71	92.95	/	/

^a^ The genome coverage is calculated based on the sesame genome size of 354 Mb estimated by K-mer [2], ^b^ For the genome sequences of 131 F_6_ progeny, the genome coverage per progeny is 16.17 fold, ^c^ Unique variants in a parent after compared with the other parent.

## Supplementary Materials

Supplementary materials can be found at https://www.mdpi.com/1422-0067/20/16/4056/s1.

## Abbreviations

AS-PCR	allele-specifific PCR
CKs	cytokinins
CL	capsule length
CRC	CRABS CLAW
cZ	cis-zeatin
czR	cis-zeatin riboside
DEGs	differentially expressed genes
DHZ	dihydrozeatin
EMS	Ehylmethane sulfonate
FC	fold change
FDR	false discovery rate
FKPM	the fragments per kilobase of transcript per million
GA	gibberellins
IAA	indole-3-acetic acid
InDel	Insertion-deletion
iP	isopenteny ladenine
iPR	isopenteny ladenosine
KEGG	Kyoto encyclopedia of genes and genomes
MT	mutant type
NGS	Next-generation sequencing technology
NR	Non- redundant
qRT-PCR	Quantitative real time PCR
QTL	Quantitative trait loci
RIL	Recombinant inbred line
SNC	Seed number per capsule
SNP	Single nucleotide polymorphism
tZ	trans-zeatin
tzR	trans-zeatin riboside
WT	wild type
