# Supplementary material for: A SNP Mutation of SiCRC Regulates Seed Number Per Capsule and Capsule Length of cs1 Mutant in Sesame
Source: Int J Mol Sci. 2019 Aug 20;20(16):4056. doi: 10.3390/ijms20164056 (PMC6720709; doi:10.3390/ijms20164056)
Supplement: Supplementary file 1 [file ijms-20-04056-s001.zip › ijms-543758 sp revised 8.12 original/ijms-543758 sp Table and figure captions.pdf]

Table S1. Phenotypic comparison of mutant type *cs1* and the wild type Yuzhi 11.

Table S2. Information of 56 pathways in DEGs analysis.

Table S3. Information of the DEGs involved in plant hormone signal transduction pathways.

Table S4. Variant information of the candidate interval controlling the *cs1* mutant trait in sesame.

Table S5. Variants information of 620 sesame germplasm accessions in target region.

Table S6. Information of candidate variants screened using genome resequencing data of 620 sesame germplasm accessions.

Table S7. Information of the 3 candidate variants for *cs1* mutant trait in sesame.

Table S8. The information of the SicsSNP marker.

Table S9. The information of the CDs and qRT-PCR marker.

Table S10. Mapping location comparison for CL and SNC traits among different studies.

Figure S1. Phenotypic comparison of mutant type *cs1* and the wild type Yuzhi 11.

Figure S2. Pearson correlation analysis of WT and MT samples.

Figure S3. DEGs analysis between WT and MT samples.

Figure S4. Amplification validation of the SicsSNP marker using the test population and germplasm accessions. M: DNA marker; Lane 1–10: F_7 lines with MT; Lane 11- 20: F_7 lines with WT; Lane 21- 40: 20 sesame accessions with WT.

Figure S5. Protein sequence comparison of SiCS1 and SiCS2 homologs in sesame.

Figure S6. Phylogeny analyses of SiCS1 protein in sesame and other plants.
