# Supplementary figures and images for: A SNP Mutation of SiCRC Regulates Seed Number Per Capsule and Capsule Length of cs1 Mutant in Sesame
Source: Int J Mol Sci. 2019 Aug 20;20(16):4056. doi: 10.3390/ijms20164056 (PMC6720709; doi:10.3390/ijms20164056)

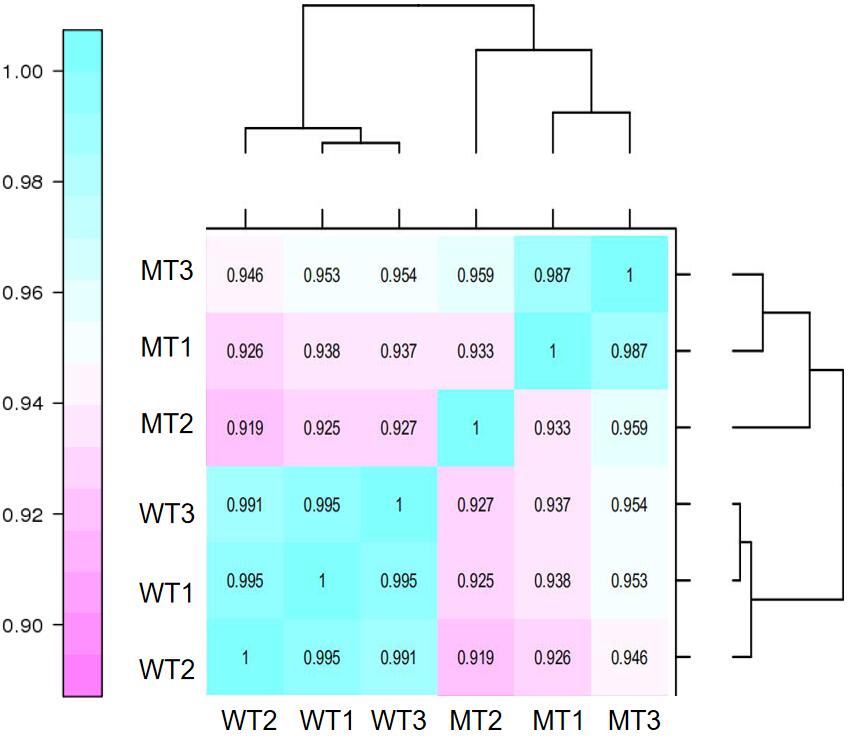

Supplement: Supplementary file 1 [file ijms-20-04056-s001.zip › ijms-543758 sp revised 8.12 original/Supplemental folder/Figure S2.jpg]

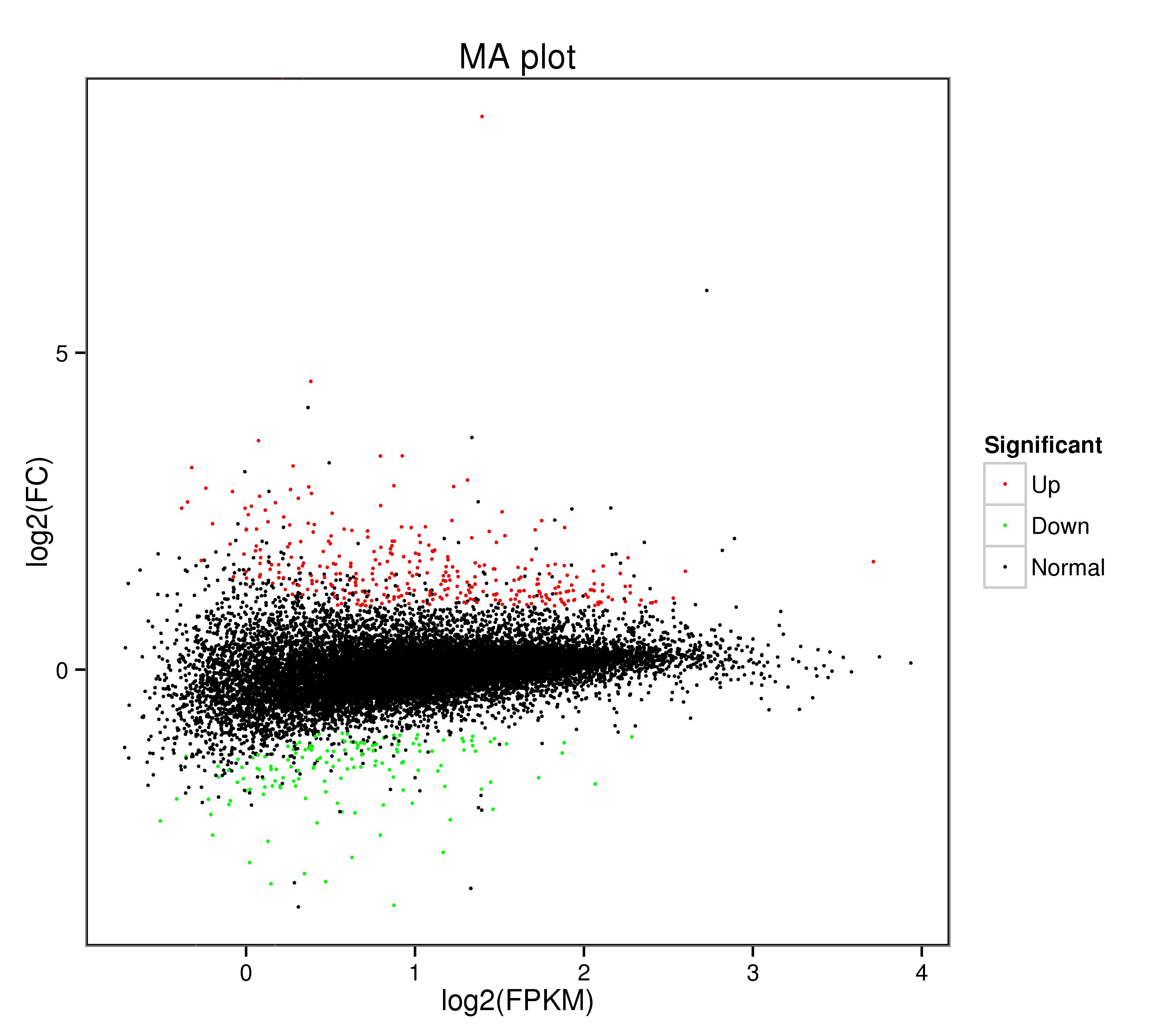

Supplement: Supplementary file 1 [file ijms-20-04056-s001.zip › ijms-543758 sp revised 8.12 original/Supplemental folder/Figure S3.jpg]

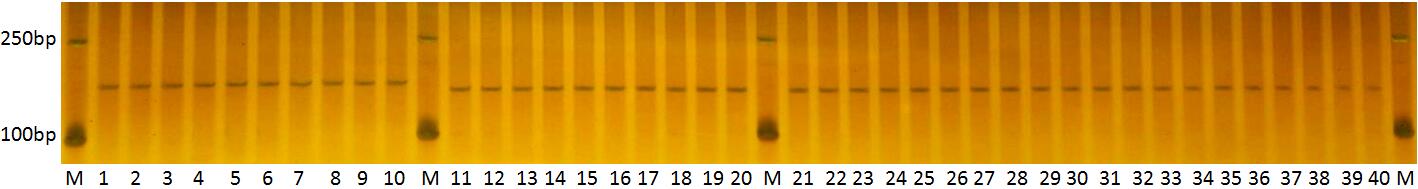

Supplement: Supplementary file 1 [file ijms-20-04056-s001.zip › ijms-543758 sp revised 8.12 original/Supplemental folder/Figure S4.jpg]

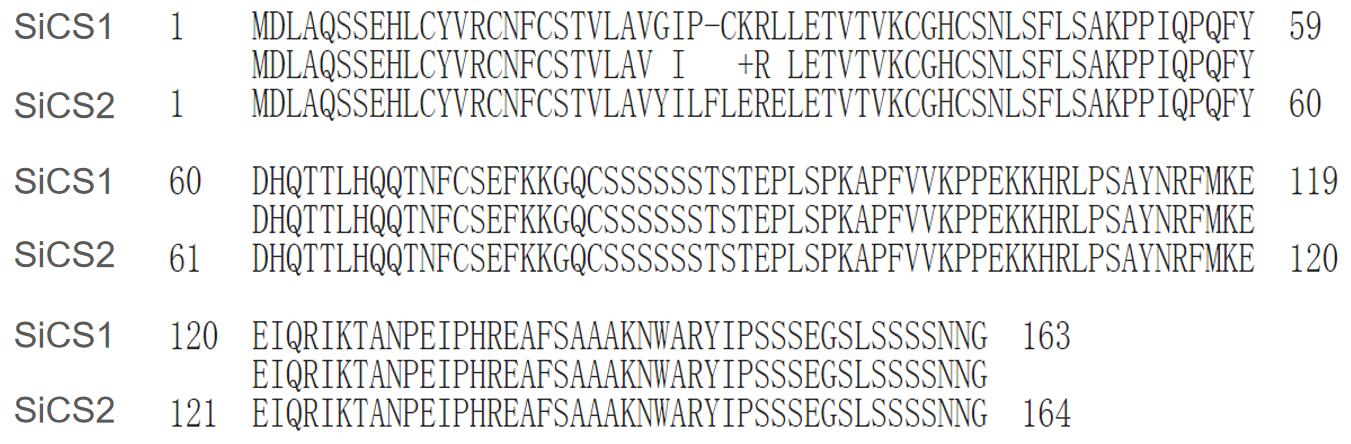

Supplement: Supplementary file 1 [file ijms-20-04056-s001.zip › ijms-543758 sp revised 8.12 original/Supplemental folder/Figure S5.jpg]

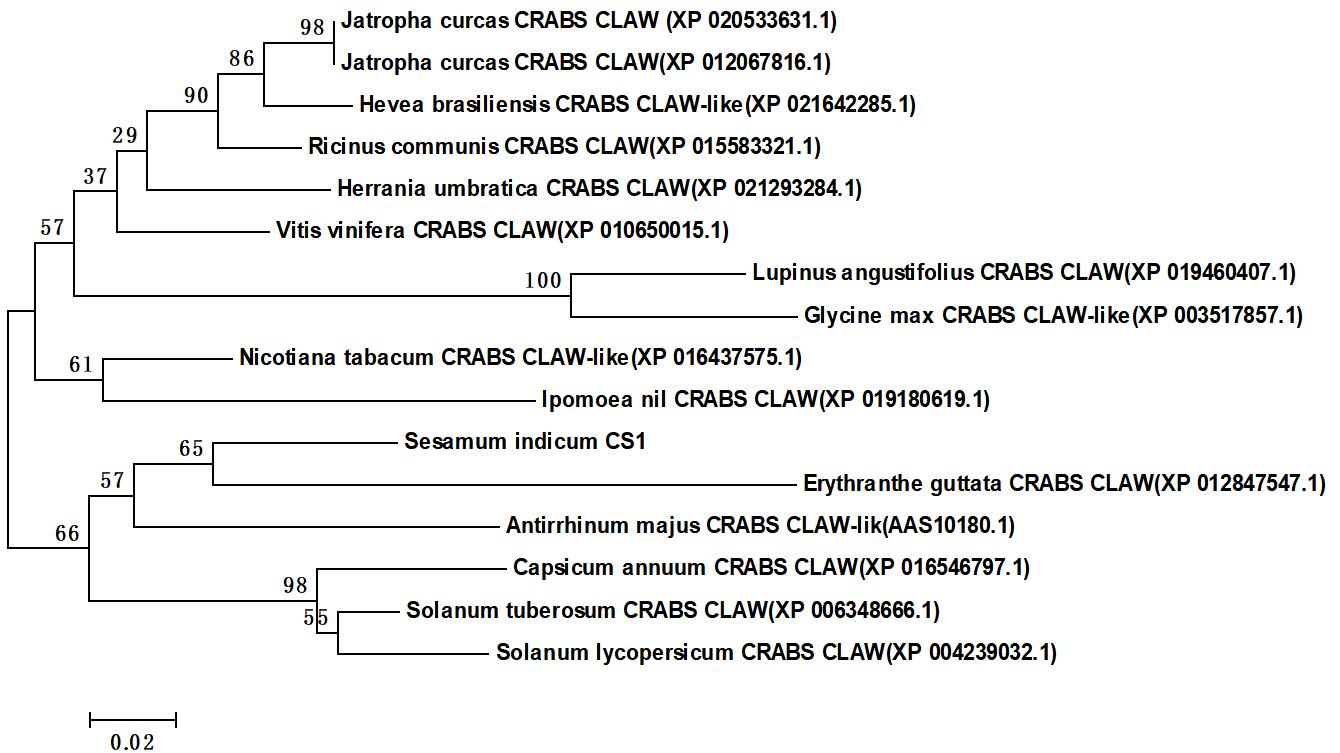

Supplement: Supplementary file 1 [file ijms-20-04056-s001.zip › ijms-543758 sp revised 8.12 original/Supplemental folder/Figure S6.jpg]

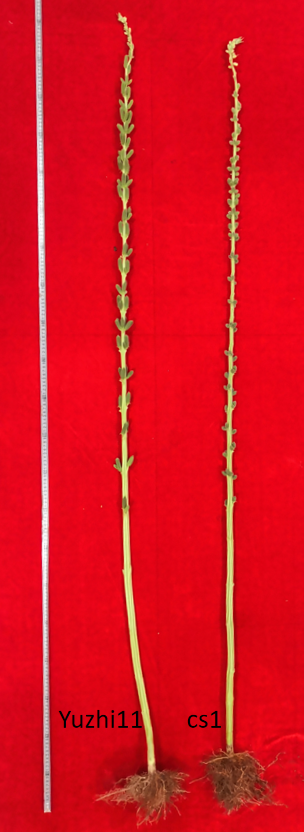

Supplement: Supplementary file 1 [file ijms-20-04056-s001.zip › ijms-543758 sp revised 8.12 original/Supplemental folder/Figure S1.tif]
